# Key Population Hotspots in Nigeria for Targeted HIV Program Planning: Mapping, Validation, and Reconciliation

**DOI:** 10.2196/25623

**Published:** 2021-02-22

**Authors:** Julia Lo, Samuel U Nwafor, Amee M Schwitters, Andrew Mitchell, Victor Sebastian, Kristen A Stafford, Idoteyin Ezirim, Man Charurat, Anne F McIntyre

**Affiliations:** 1 Institute of Human Virology University of Maryland School of Medicine Baltimore, MD United States; 2 Center for International Health, Education, and Biosecurity University of Maryland School of Medicine Baltimore, MD United States; 3 Center for International Health, Education, and Biosecurity University of Maryland School of Medicine Abuja Nigeria; 4 Division of Global HIV & TB Center for Global Health Centers for Disease Control and Prevention Abuja Nigeria; 5 Department of Epidemiology and Public Health University of Maryland School of Medicine Baltimore, MD United States; 6 National Agency for the Control of AIDS Abuja Nigeria; 7 Division of Global HIV & TB Center for Global Health Centers for Disease Control and Prevention Atlanta, GA United States

**Keywords:** key population, female sex workers, men who have sex with men, people who inject drugs, hotspot mapping, HIV, Nigeria

## Abstract

**Background:**

With the fourth highest HIV burden globally, Nigeria is characterized as having a mixed HIV epidemic with high HIV prevalence among key populations, including female sex workers, men who have sex with men, and people who inject drugs. Reliable and accurate mapping of key population hotspots is necessary for strategic placement of services and allocation of limited resources for targeted interventions.

**Objective:**

We aimed to map and develop a profile for the hotspots of female sex workers, men who have sex with men, and people who inject drugs in 7 states of Nigeria to inform HIV prevention and service programs and in preparation for a multiple-source capture-recapture population size estimation effort.

**Methods:**

In August 2018, 261 trained data collectors from 36 key population–led community-based organizations mapped, validated, and profiled hotspots identified during the formative assessment in 7 priority states in Nigeria designated by the United States President’s Emergency Plan for AIDS Relief. Hotspots were defined as physical venues wherein key population members frequent to socialize, seek clients, or engage in key population–defining behaviors. Hotspots were visited by data collectors, and each hotspot’s name, local government area, address, type, geographic coordinates, peak times of activity, and estimated number of key population members was recorded. The number of key population hotspots per local government area was tabulated from the final list of hotspots.

**Results:**

A total of 13,899 key population hotspots were identified and mapped in the 7 states, that is, 1297 in Akwa Ibom, 1714 in Benue, 2666 in Cross River, 2974 in Lagos, 1550 in Nasarawa, 2494 in Rivers, and 1204 in Federal Capital Territory. The most common hotspots were those frequented by female sex workers (9593/13,899, 69.0%), followed by people who inject drugs (2729/13,899, 19.6%) and men who have sex with men (1577/13,899, 11.3%). Although hotspots were identified in all local government areas visited, more hotspots were found in metropolitan local government areas and state capitals.

**Conclusions:**

The number of key population hotspots identified in this study is more than that previously reported in similar studies in Nigeria. Close collaboration with key population–led community-based organizations facilitated identification of many new and previously undocumented key population hotspots in the 7 states. The smaller number of hotspots of men who have sex with men than that of female sex workers and that of people who inject drugs may reflect the social pressure and stigma faced by this population since the enforcement of the 2014 Same Sex Marriage (Prohibition) Act, which prohibits engaging in intimate same-sex relationships, organizing meetings of gays, or patronizing gay businesses.

## Introduction

Key populations include female sex workers, men who have sex with men, and people who inject drugs, and they are particularly vulnerable to HIV. The high incidence and prevalence of HIV in key populations are well documented in the literature [1-7]. Together, key population members and their sexual partners account for 54% of the new HIV infections worldwide, with the risk of HIV acquisition being up to 22-fold higher for female sex workers, men who have sex with men, and people who inject drugs than the risk of HIV acquisition by the general population [8]. Stigma and discrimination, fear of legal prosecution, misinformation, travel time, and transportation costs are some of the barriers to HIV testing and treatment among the general population and key population members alike [9-14].

Nigeria is characterized as having a mixed HIV epidemic with high HIV prevalence among key population members and low prevalence in the general population [15,16]. Recently published results from the Nigeria HIV/AIDS Indicator and Impact Survey revealed an HIV prevalence of 1.4% among men and women in the age range of 15-49 years, which is considerably lower than previous estimates [16,17]. In contrast, the 2014 data from the most recent biobehavioral survey among key populations demonstrated HIV prevalence of 14.4% among female sex workers, 22.9% among men who have sex with men, and 3.4% among people who inject drugs [15]. Key populations and their sexual partners are estimated to account for 32% of the new HIV infections in Nigeria [18]. Thus, targeted interventions designed to serve and reach these individuals become a necessary strategy to control the HIV epidemic. In the National HIV and AIDS Strategic Framework 2017-2021, the Government of the Federal Republic of Nigeria outlined the need for interventions to increase testing and treatment for key populations in order to fast-track the national response toward ending AIDS in Nigeria by 2030 [18,19]. Reliable and accurate information on where key population members socialize is needed for the strategic placement of services and allocation of limited resources for targeted interventions.

It is recommended that key population mapping and population size estimation be conducted every 2-3 years to produce reliable and up-to-date data for HIV program planning [20]. There have been several efforts to map key population hotspots in Nigeria in 2009, 2013, and 2015 [21-23]. Several of these studies had shortcomings, including failure to report the number of hotspots of men who have sex with men identified and limitations in scope, reporting only on male sex workers and only in major cities. The objective of this study was to map the key population hotspots in 7 United States President’s Emergency Plan for AIDS Relief (PEPFAR) priority states in Nigeria. These states were chosen in consultation with the Government of the Federal Republic of Nigeria and others due to the evidence of high HIV burden and unmet needs for HIV/AIDS treatment services at the time this study was conducted [24]. This study is the first of a two-part key population mapping and size estimation effort. The scope of this paper is to report on the mapping exercise only. The multiple-source capture-recapture (MS-CRC) population size estimation exercise is a separate report. The final list of the key population hotspots generated from this exercise served as a sampling frame for MS-CRC.

## Methods

### Study Sites

This study was conducted in 7 PEPFAR priority states of Akwa Ibom, Benue, Cross River, Lagos, Nasarawa, Rivers, and Federal Capital Territory and as part of a larger key population hotspot mapping and size estimation exercise that included a formative assessment and MS-CRC for population size estimation. This exercise was conducted in all 134 local government areas in the 7 states.

### Study Procedures

For planning and logistical purposes, each state was divided into 3 smaller geographical areas, referred to as zones. Prior to the start of the field activities, a list of known hotspots of female sex workers, men who have sex with men, and people who inject drugs was compiled from stakeholders, including Nigeria National Agency for Control of AIDS (NACA) and implementing partners who provide services to key population members or were involved in previous key population hotspot mapping exercises (Society for Family Health, 2015) [23]. This list was stratified by study states and zones and reviewed during state-specific and zone-specific focus group discussions and key informant interviews as part of the formative assessment. During the formative assessment, additional key population hotspots were added to the list and those reported to be inactive were marked but not deleted.

In August 2018, 261 trained data collectors in the 7 states mapped, validated, and profiled the hotspots collated at the end of the formative assessment. During mapping, data collectors used tablets to electronically record the hotspot name, local government area, address, type, and geographic coordinates. In addition, data collectors identified 1 key informant in the area to solicit peak times of key population activity, presence of key population behaviors of interest, and an estimate of the minimum and maximum number of key population members found in the hotspot at the time of their visit. Key informants were bartenders, bouncers/security staff, madams/brothel owners, bunk owners/drug peddlers, or taxi drivers, and they displayed familiarity with key population activities in the hotspot. In addition to validating known hotspots, data collectors identified new hotspots that had emerged since the previous mapping exercise in 2015.

For this study, hotspots were defined as physical locations in which key population members frequent to socialize, seek clients, or engage in key population–defining behaviors. The following definitions of hotspot types were used in this study. Those located in an outdoor area accessible to the public were categorized as street/public place. These included streets, under bridges, organized motor parks, unnamed drinking places, and bus stops. Hostel/campus was defined as an area near student living apartments/hostels of a secondary, polytechnic, or university level academic institution meant for student relaxation, academic meetings, or social gatherings. Hotspots not captured in the categories as described in the protocol were classified as “Other.”

A desk review was conducted after data collection in the field to verify the information submitted and to remove duplicates. Hotspots were reviewed by data analysts, and potential duplicates were flagged based on similarity in name, address, geographic coordinates, and other relevant information. State supervisors then met with data collectors and representatives of the key population community to review the list of hotspots and to remove duplicate entries. We utilized this opportunity to further improve on information collected during the hotspot mapping exercise. Corrections on location information such as local government area, address, name of hotspot, and documented geocoordinates were made following suggestions from data collectors during MS-CRC. Information on additional duplicates was also solicited for documentation and flagged but not removed. No additional hotspots were added or removed from the initial list of hotspots at the start of MS-CRC.

### Technical Team in This Study

A national technical team was formed to support and inform study design, implementation, monitoring, and dissemination. The national technical team, chaired by NACA, was composed of members from the Nigeria Federal Ministry of Health, the University of Maryland Baltimore, the US Centers for Disease Control and Prevention (CDC) in Nigeria, the National Key Affected Population Network, Centre for the Right to Health, Heartland Alliance International Nigeria, and Population Council. In addition to the national technical team, a state technical team was formed in each of the 7 study states to guide implementation, ensure security, provide close monitoring, and increase buy-in at the state level. Similar to the national technical team, the state technical teams were composed of representatives from the government, key population community, implementing partners who provide services to key population, and University of Maryland Baltimore, in addition to representatives from the Police Action Committee on HIV/AIDS or National Drug Law Enforcement Agency, to help ensure the safety of the data collectors.

### Local Partnerships and Recruitment of Data Collectors

In each state, we engaged a minimum of 3 key population–affiliated community-based organizations, one for each key population group, to help implement and inform study planning and logistics. Most data collectors for the exercise were key population members and were recruited through local partner key population community-based organizations. The few data collectors who did not identify as key population members had experience working with key population members and were trusted within the local key population community. While recruiting data collectors for this exercise, the study team considered the data collector’s level of influence within the key population community in facilitating hotspot access for the study team, familiarity with key population networks and hotspots, experience in previous surveys, service delivery, or research activities, and considered diversity in age, base of operation (eg, brothel-based or street-based female sex workers), geographical area of familiarity, and other relevant experience. Data collectors were essential in facilitating access to hotspots, and diversity proved to be especially valuable in facilitating access to hotspots frequented by minority key population groups, for example, older female sex workers. Following advice from local partners to prioritize safety and acceptance of the mappers, data collection teams were assigned to map hotspot types that aligned with the key population identity of the team members. For example, hotspots frequented by people who inject drugs were assigned to teams composed of data collectors collecting data on people who inject drugs. A total of 36 key population community-based organizations and 261 data collectors were formally engaged in the 7 states. Most data collectors were retained for the population size estimation component of the study following this hotspot mapping exercise.

### Data Management and Quality Assurance

Hotspot information was collected using REDCap (Research Electronic Data Capture) data collection and management software [25,26]. Skip logic and data validation rules were built-in throughout the data collection tool to prevent entry of implausible data. All data collectors were trained in data entry using REDCap, study objectives, standard operating procedures, and the importance of discretion and confidentiality. Data monitoring officials and state supervisors observed the uploaded information to check for missing data fields and inconsistent or implausible values. In addition, members of the national technical team and state technical teams conducted field visits to ensure that data collectors were capturing project data according to study procedures.

### Data Analysis and Map Development

The number of key population hotspots per local government area was tabulated from the final list of hotspots, and the number of hotspots per 100,000 population was calculated using 2016 population projections by local government area [27]. The latter was done to allow for comparison of the number of hotspots across local government areas independent of the population size of the local government area. The number of key population hotspots per 100,000 population by local government area was mapped using ArcMap 10.5.1 (Esri). For clarity without crowding the maps, quintiles were used to display differences in the number of hotspots per 100,000 population across the local government areas in the 7 states. State-level distributions of the hotspot types for each key population group were tabulated from the final list of hotspots. Data cleaning and basic analyses as described above (tabulation, determining mean, median, and mode, calculation of rates, and cutoff points for maps) were performed using Stata 15 (StataCorp LLC).

### Research Protection of Human Subjects

This study was approved by the National Health Research Ethics Committee, Nigeria and the Institutional Review Board of University of Maryland Baltimore. This study was reviewed in accordance with the US CDC human research protection procedures and determined to be research, but CDC investigators did not interact with the human subjects or have access to identifiable data for research purposes.

## Results

**Figure 1:**
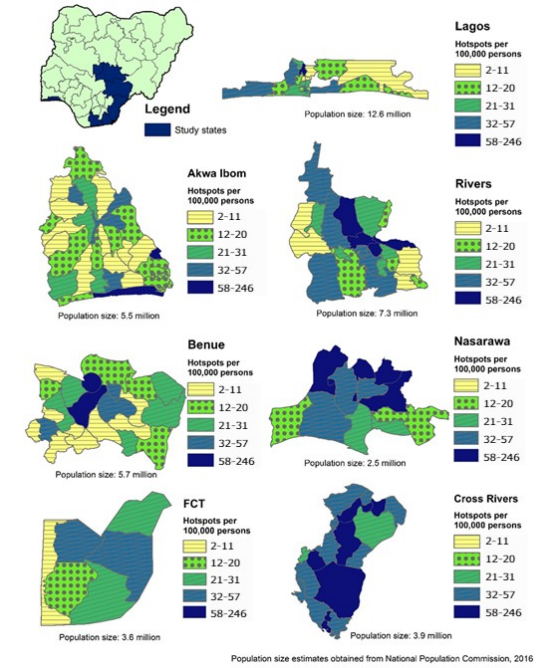
Number of key population hotspots per 100,000 population by local government area in 7 US President’s Emergency Plan for AIDS Relief Priority States in Nigeria.

A total of 13,899 key population hotspots were mapped and identified in the 7 states. States with the largest number of hotspots were Lagos, Cross River, and Rivers (Table 1). Although more hotspots were found in urban local government areas and state capitals, at least one was identified in each of the 134 local government areas visited (Figure 1). In Figure 1, the number of key population hotspots presented in the map represents findings at the end of hotspot mapping and validation, immediately before MS-CRC activities began. Hotspots were dynamic with frequent changes in activity status. Hotspot mapping, validation, and data reconciliation were performed between August 2018 and December 2018. The local government areas of key population hotspots were indicated by enumerators, with maps representing the number of key population hotspots per 100,000 population per local government area. Population estimates by local government area [27] were obtained on March 13, 2019. The shape files of Nigerian geographic boundaries [28] were obtained from the Office of the Surveyor General of the Federation, eHealth, United Nations Cartographic Section on February 23, 2017. Among the 134 local government areas, all had at least one female sex worker hotspot, 129 (96.3%) areas had at least one hotspot of people who inject drugs, and 118 (88.1%) areas had at least one hotspot of men who have sex with men. The number of hotspots found in each state and local government area varied by key population group and depended largely on the size of the general population. Compared to hotspots frequented by female sex workers and people who inject drugs, far fewer were found for men who have sex with men (Table 1). The number of hotspots mapped during our 2018 study is summarized with the results from the previous mapping exercises in 2013 and 2015 for comparison of the coverage by key population and state (Table 1).

**Table 1:** Number of hotspots identified in 7 US President’s Emergency Plan for AIDS Relief priority states in Nigeria by key population in 2013, 2015, and 2018.

State	Female sex worker hotspots (n)				Men who have sex with men hotspots (n)				People who inject drugs hotspots (n)				Total hotspots (N)		
	2013^a^	2015^b^	2018	2013		2015	2018	2013		2015	2018	2013		2015	2018
Akwa Ibom	—^c^	150	708	—		—	276	—		89	313	—		239	1297
Benue	825	344	1098	57		—	265	32		117	351	914		461	1714
Cross River	692	497	1782	15		—	268	8		192	616	715		689	2666
Federal Capital Territory	1446	677	977	120		—	116	22		41	111	1588		718	1204
Lagos	4056	2534	2603	191		—	131	95		230	240	4342		2764	2974
Nasarawa	1409	575	990	19		—	246	12		375	314	1440		950	1550
Rivers	—	393	1435	—		—	275	—		141	784	—		534	2494
Total	8428	5170	9593	402		—	1577	169		1185	2729	8999		355	13,899

^a^Source for 2013 data [22].

^b^Source for 2015 data [23].

^c^Not available.

**Figure 2:**
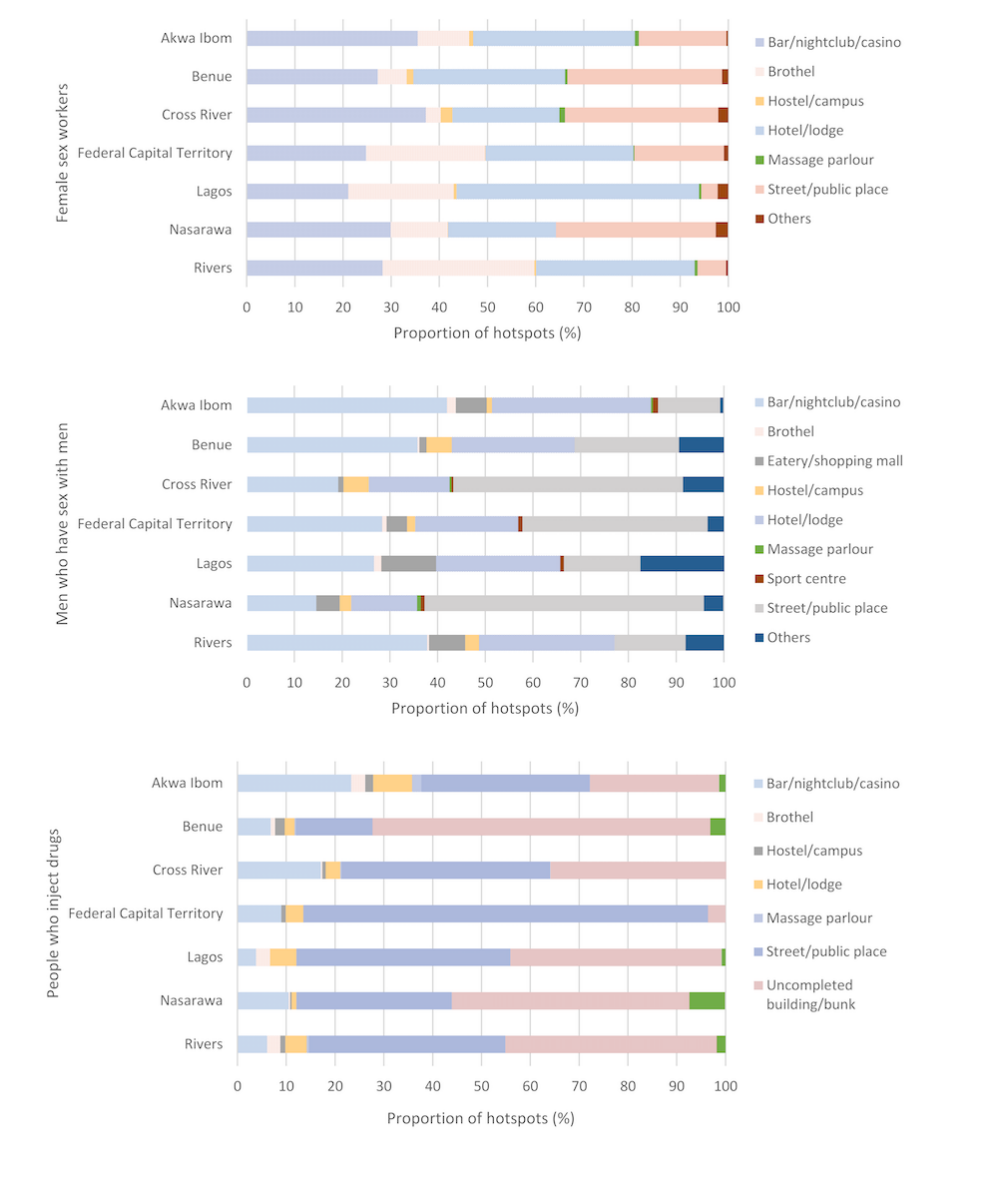
Type of key population hotspots by state.

Of all the female sex worker hotspots mapped in the 7 states, the majority (9253/9548, 96.9%) of the hotspot types fell into 1 of the following 4 categories: hotel (3266/9548, 34.2%), bar/nightclub/casino (2693/9548, 28.2%), street/public place (18.1%), and brothel (1569/9548, 16.4%). Popular categories of men who have sex with men hotspots in our study included street/public place (473/1575, 30.0%), bar/nightclub/casino (470/1575, 29.8%), and hotel/lodge (376/1575, 23.9%). Of the hotspots of people who inject drugs, 42.1% (1144/2718) mapped in our study were categorized as uncompleted buildings/bunks, 38.1% as street/public place, and 11.1% as bar/nightclub/casino. A small proportion of hotspots mapped in the 7 states were of unknown type and are not reflected in the proportions listed above. These included 0.5% (45/9953) of female sex worker hotspots, 0.1% (2/1577) of men who have sex with men hotspots, and 0.4% (11/2729) of people who inject drugs hotspots. The most popular type of hotspot varied by state and key population group (Figure 2).

## Discussion

### Overview of Our Findings

A total of 13,899 key population hotspots in 7 states were identified; these hotspots are more than the 6355 total hotspots previously reported from the last mapping exercise in the same 7 states of Nigeria during 2015 [22,23]. The sizable differences in the number of hotspots may be explained, in part, by divergent methods. Similar strategies were used to map hotspots of female sex workers and people who inject drugs in 2015 and 2018. We applied those strategies to map hotspots of men who have sex with men in 2018 as well. However, the 2015 efforts did not include hotspot mapping of men who have sex with men because investigators were concerned about the poor social visibility, and they focused efforts on capture-recapture to identify men who have sex with men for population size estimation. Although there might be additional unknown factors that contributed to identifying more hotspots in 2018 than in 2015, community engagement played a major role in our efforts. Our strong collaboration with local key population–affiliated communitybased organizations facilitated entry into the key population community and enabled us to identify many new and previously undocumented hotspots. The diversity of the data collectors recruited enabled us to identify more hotspots frequented by minority key population groups, for example, older key population members or female people who inject drugs, in addition to many hotspots that were more commonly known among the public and the key population community.

We found fewer hotspots frequented by men who have sex with men compared to those frequented by female sex workers or people who inject drugs, and our data supported the notion that unlike female sex workers and people who inject drugs, public hotspots specific to men who have sex with men were less common, and based on information learned during the field activities from community members, this group tended to gather in private residences. This may be attributed to the widespread stigma against men who have sex with men in Nigeria that has made community members more reluctant to congregate in overt settings. We have previously described an increase in stigma following the enforcement of the 2014 Same Sex Marriage (Prohibition) Act, which made it “illegal not only to engage in an intimate relationship with a member of the same sex, but to attend or organize a meeting of gays, or patronize or operate any type of gay organization, including private clubs” [29-32]. Violence by the public and police against members of the lesbian, gay, bisexual, and transgender community was widely reported following the passing of the Same Sex Marriage (Prohibition) Act causing many lesbian, gay, bisexual, and transgender members, including men who have sex with men, to go into hiding [32,33].

Overall, hotspots of men who have sex with men and people who inject drugs were more difficult to locate compared to female sex worker hotspots and often required the knowledge of local key population informants. It is often advantageous for female sex worker hotspots to be visible and known to the public as a way for female sex workers to solicit their clients. The same concept does not apply for men who have sex with men and persons who inject drugs because of lower social acceptability and the potential for increased risk to their safety, although knowledge of male sex worker hotspots may be more common in the community of men who have sex with men. Although we report finding fewer hotspots of men who have sex with men and people who inject drugs than those of female sex workers, the absolute number of hotspots of men who have sex with men and people who inject drugs identified in this study is larger than that previously documented in Nigeria; 402 hotspots of men who have sex with men were reported by NACA in 2013 in the 5 states of Benue, Cross River, Lagos, Nasarawa, and Federal Capital Territory compared to 1026 hotspots of men who have sex with men that we report here (the Society for Family Health did not report the number of hotspots of men who have sex with men that they found in 2015) and 1184 hotspots of people who inject drugs were reported by the Society for Family Health in 2015 compared to 2729 hotspots of people who inject drugs that we report here [22,23].

We report more key population hotspots in state capitals and metropolitan city centers compared to those in less developed and metropolitan local government areas. This is true even after adjusting for the population size in the local government area. This finding is consistent with key population hotspot mapping and size estimation exercises conducted previously in Nigeria and in other countries [34]. This finding is also consistent with the expectations and experiences among stakeholders in the country and previous studies conducted in Nigeria [21-23]. Of the states in our study, Lagos was the most developed, commercial, and populous state with a projected population of 12.6 million. In comparison, our second, third, and fourth most populous states, that is, Rivers, Benue, and Akwa Ibom, had projected populations of 7.3 million, 5.7 million, and 5.5 million, respectively [27]. The projected population (2016) for Nigeria is 193 million [27]. Comparing the total number of hotspots by state with results published by the Society for Family Health in 2015, the largest difference in the number of hotspots of female sex workers and those of people who inject drugs were in Akwa Ibom, Benue, Cross River, and Rivers [23].

Our findings have important programming and policy implications. In addition to the substantially larger number of hotspots of female sex workers and people who inject drugs identified during 2018 compared to that identified in 2015, we also mapped the hotspots of men who have sex with men, filling a large data gap in Nigeria. Local government area health authorities are best positioned to identify other possible factors such as structural and behavioral changes that might have contributed to the growth of key population hotspots since 2015. Data may be used as is or linked by geocodes with a variety of other data sources (eg, health care facilities, testing sites, pharmacies, census projections, economic indicators) to inform outreach and prevention efforts, subnational HIV program target setting, or evaluation of service coverage and utilization. All results should be examined in the context of current HIV prevention programs and service delivery to ensure that response efforts are aligned and scaled appropriately to maximize impact and cost-effectiveness. Resources might need to be reallocated to broaden outreach and service coverage to include the newly identified hotspots. Hotspot profiles, including typology and peak activity, will assist in customizing efficient service delivery on the highest impact days and times at hotspots to reach specific subgroups within each key population. Our findings also provide rationale for increased advocacy for key population policy changes to reduce stigma and discrimination that might be hindering HIV response efforts.

These findings can be used by HIV service providers to identify strategic areas for placement of services and allocation of limited resources for targeted interventions. In addition to stigma and discrimination by health workers, travel time and transportation costs to treatment centers have been cited as barriers to treatment access and retention [9-14]. Placing prevention and treatment services that are conveniently located to venues where key populations socialize, seek clients, or inject drugs could increase testing and retention rates among key population members. Venue-based HIV testing and prevention services were successful in reaching female sex workers and men who have sex with men who were unaware of their status in Malawi and Angola; 71% were not previously aware of their HIV status and received a diagnosis through the study [35]. Other studies report similar findings, supporting the success of venue-based testing and prevention services in reaching high-risk groups [36-39].

We engaged the key population community during formative assessment and continued partnership with key population community-based organizations throughout our activities. This effort proved to be enormously helpful in facilitating entrance to the key population community and activity buy-in. Close collaboration with local stakeholders, including government entities, law enforcement, and key population community-based organizations, minimized adverse outcomes from several security challenges during field activities. Our strong partnership with the local key population community-based organizations enabled us to identify more hotspots and strengthened partnerships for future collaboration with HIV programs.

This study was conducted as part of a larger key population hotspot mapping and size estimation activity that included an MS-CRC for population size estimation. The final list of hotspots produced from this exercise was used as the sampling frame for MS-CRC. MS-CRC also provided an opportunity to further correct and improve on hotspot location information such as local government area, address, and name of hotspot. Accurate documentation of addresses was essential as the list was used by different groups of enumerators to locate the same hotspot during MS-CRC.

### Challenges and Limitations of This Study

The lack of formal addresses in our setting and multiple (slang) names for hotspots made de-duplication and locating of hotspots difficult. Although our partnership with the local stakeholders minimized security incidents and other challenges, there were still several security issues (eg, hotspots in or near areas where kidnapping, militancy, or other unrest were threats or travel to very remote hotspots requiring long drives into the night or an hour of water transportation via sea) and inclement weather conditions (ie, seasonal rains) during the data collection period that rendered several hotspots inaccessible. After fieldwork, the study team conducted additional reviews to verify and de-duplicate the list of key population hotspots. Our close partnerships with local key population community-based organizations were critical in this process. Although extensive time and resources were dedicated to de-duplicate, locate, and validate all hotspots, duplicates and inaccuracies may still be present.

As key population–defining behaviors are illegal in Nigeria and key population members often must evade law enforcement, the closing and relocation of hotspots were common. The numbers presented here reflect the situation at the time of our fieldwork. The states where our study was conducted were chosen as they were PEPFAR priority states at the time this study was conducted with evidence of high HIV burden and unmet needs for HIV/AIDS treatment services. These results should not be generalized to other states in Nigeria.

### Conclusions

We identified many new and previously undocumented key population hotspots in the 7 priority states in Nigeria designated by the United States President’s Emergency Plan for AIDS Relief. The implications of our findings have broad impact. Not only do they highlight the need for these states to strategically position and scale-up outreach and service programs, but also emphasize the need to effectively combat stigma and discrimination if Nigeria aims to have a fully successful HIV response. Engaging local key population community-based organizations throughout this activity allowed identification of a larger number of hotspots and strengthened partnerships for future collaboration with HIV programs. The small number of hotspots of men who have sex with men compared to that of female sex workers and people who inject drugs might be attributed to the 2014 Same Sex Marriage (Prohibition) Act or the practice of retreating into private residences, reflecting the low social visibility among these groups. Future studies may wish to consider broadening their definition of hotspots of men who have sex with men to include events in private residences or even web-based social platforms, which were increasing in popularity among the key populations in our setting. The information obtained from this exercise is expected to be used by the Government of the Federal Republic of Nigeria, donors, and implementing partners to design more strategically located and appropriately scaled key population–specific interventions. In addition, the final list of hotspots produced from this exercise was used as a sampling frame for MS-CRC for population size estimation, which was performed between October 2018 and December 2018 and summarized in a separate report.

## Abbreviations

CDC: Centers for Disease Control and Prevention
MS-CRC: multiple-source capture-recapture
NACA: National Agency for Control of AIDS
PEPFAR: President’s Emergency Plan for AIDS Relief
REDCap: Research Electronic Data Capture
